# A Noisy SAR Image Fusion Method Based on NLM and GAN

**DOI:** 10.3390/e23040410

**Published:** 2021-03-30

**Authors:** Jing Fang, Xiaole Ma, Jingjing Wang, Kai Qin, Shaohai Hu, Yuefeng Zhao

**Affiliations:** 1 Shandong Province Key Laboratory of Medical Physics and Image Processing Technology, School of Physics and Electronics, Shandong Normal University, Jinan 250014, China; fangjing@sdnu.edu.cn (J.F.); wjj@sdnu.edu.cn (J.W.); 2 Shandong Provincial Engineering and Technical Center of Light Manipulations & Shandong Provincial Key Laboratory of Optics and Photonic Device, School of Physics and Electronics, Shandong Normal University, Jinan 250014, China; 3 Institute of Information Science, Beijing Jiaotong University, Beijing 100044, China; maxiaole@bjtu.edu.cn; 4 School of Environment Science and Spatial Informatics, China University of Mining and Technology, Xuzhou 221116, China; qinkai@cumt.edu.cn

**Keywords:** nonlocal matching, generative adversarial networks, image fusion

## Abstract

The unavoidable noise often present in synthetic aperture radar (SAR) images, such as speckle noise, negatively impacts the subsequent processing of SAR images. Further, it is not easy to find an appropriate application for SAR images, given that the human visual system is sensitive to color and SAR images are gray. As a result, a noisy SAR image fusion method based on nonlocal matching and generative adversarial networks is presented in this paper. A nonlocal matching method is applied to processing source images into similar block groups in the pre-processing step. Then, adversarial networks are employed to generate a final noise-free fused SAR image block, where the generator aims to generate a noise-free SAR image block with color information, and the discriminator tries to increase the spatial resolution of the generated image block. This step ensures that the fused image block contains high resolution and color information at the same time. Finally, a fused image can be obtained by aggregating all the image blocks. By extensive comparative experiments on the SEN1–2 datasets and source images, it can be found that the proposed method not only has better fusion results but is also robust to image noise, indicating the superiority of the proposed noisy SAR image fusion method over the state-of-the-art methods.

## 1. Introduction

As one of the active microwave imaging radars, synthetic aperture radar (SAR) can work at any time and in any weather conditions. The many advantages of SAR includes, among others, multi-polarization and variable angles, which allows SAR images to be widely used in geological surveys, military exercises, etc. [1,2]; however, due to its special coherent imaging mechanism, noise is inevitably generated in image acquisition, especially for speckle noise, resulting in serious inconvenience to the subsequent interpretation of the image processing; therefore, the effective suppression or removal of noise is one of the essential tasks required for SAR image pre-processing [3]. SAR can penetrate the earth’s surface as well as natural vegetation coverings, clearly and exhaustively map topography and geomorphology, and obtain high-resolution images of the earth’s surface; however, the color information of SAR images is relatively simple, and cannot adequately reflect the scene’s spectral information. On the contrary, multi-spectral sensors can obtain images with rich spectral information, such as color optical images [4]. Image fusion [5,6,7] is a powerful image processing tool for integrating complementary information from different sensors, by which a fused image with a more comprehensive and clearer description of the scene can be obtained. Although an increasing number of papers about image fusion are published every year—indicating the importance of image fusion—few papers are published regarding noisy SAR image fusion, despite the urgent need for an effective and practical SAR image fusion method.

Image fusion can be classified into pixel-level fusion, feature-level fusion, and decision-level fusion [8,9]. Pixel-level fusion fuses the pixels of source images directly, which is the basis of other level fusions; however, a significant amount of information has to be processed. Feature-level fusion extracts feature information of images such as edge, shape, texture, etc., then fuses them together; because it only extracts features for image fusion, detailed information is often missing. The most advanced decision-level fusion is based on feature-level fusion. After feature extraction, other image processing methods, including classification, recognition, and comprehensive evaluation, are employed to make a final decision. This kind of method is based on a cognitive model, which needs large databases and expert decisions for analysis.

Normally, pixel-level image fusion methods can be classified into five categories [6], including methods based on spatial domain, methods based on transform domain, methods based on sparse representation, methods based on deep learning, and hybrid methods. The methods based on spatial domain use some mathematical operations, such as “choose-max” and “average-weighting” to process the pixel value of source images directly and obtain the pixel value at the corresponding position of the fused image. The classic methods include image fusion based on maximization [10], image fusion based on intensity-hue-saturation (IHS) transform, principal component analysis (PCA) transform [11], and so on. To make full use of the image features, we employ image transforms to filter images into the feature domain. Image fusion can be realized in the transform domain. Similar to image transforms, sparse representation also transfers source images from the spatial domain to another image feature domain by the mathematical transformation; thus, this can be considered a special method based on image transform. In order to make full use of the characteristics of images in the spatial domain and the sparse features in the transform domain, many scholars mixed different kinds of image fusion methods together to obtain new image fusion methods, including image fusion based on the spatial domain and transform domain, image fusion based on the transform domain and sparse representation, and so on; however, these traditional methods usually involve fusion rule designing, which is difficult to adapt to the characteristics of different source images and cost much computing time [12].

In recent years, rapid progress has been made in deep learning, computer vision, and image processing [13], and a significant amount of research is currently being conducted in image fusion. For example, Liu [14] proposed a multi-focus image fusion method with a deep convolutional neural network; Zhong [15] proposed a remote sensing image fusion method with a convolutional neural network; Ma [12] is the first one who applied generative adversarial networks (GAN) into infrared and visible image fusion and achieved good fusion results; however, the construction of the existing FusionGAN-based method is simple, and loss fusion is imperfect, which can lead to incomplete information transfer in the fused image. Improved GAN-based methods have been proposed for image fusion, such as Zhang [16], who proposed a new generative adversarial network with adaptive and gradient joint constraints to fuse multi-focus images. Nevertheless, the methods mentioned above are effective for other kinds of source images rather than remote sensing images, and they are sensitive to the noise in an image. In this paper, we propose a noisy SAR image fusion method based on nonlocal matching (NLM) and GAN, which is more robust to noise and can serve more information from source images. The nonlocal theory is to find similar blocks in the whole image rather than in a local window and has been widely used in SAR image de-noising [17,18,19,20,21]. The nonlocal matching takes advantage of the existence of a pattern or similar features in the non-adjacent pixels and exploits self-similarities in the search neighborhood to estimate the true value of the noisy pixel. In this paper, NLM is employed on source images to acquire similar image block groups. The generator of GAN can generate a fused noise-free image block group, and the final fused image can be obtained by aggregating the blocks after the generator and discriminator are dynamically balanced. The main contributions of this paper can be summarized as follows:

(1) Due to the existence of speckle noise, SAR image de-noising is a necessary pre-processing technology; however, in our approach, we develop SAR image de-noising and fusion simultaneously, which can avoid the complex pre-processing and save more time;
(2) Nonlocal matching is employed as a pre-processing technology for GAN to obtain similar block groups, which makes full use of similar information in source images and provides more effective inputs for GAN;
(3) For image fusion, “standard, well-integrated” reference images often do not exist; i.e., when a deep learning method is used to fuse the source images, there is no reference tag; therefore, GAN is employed to perform the image de-noising and fusion without reference images by limiting the loss functions.

The rest of this paper can be summarized as follows. In Section 2, the conceptualization of GAN and its family are described. Section 3 presents the proposed noisy SAR image fusion method. Section 4 gives more details about the comparative experimental results and analysis. Finally, the conclusion is made in Section 5.

## 2. GAN

Since GAN was proposed by Ian Goodfellow in 2014 [22], it has been widely used in image processing and other fields, such as GAN-based synthetic medical image augmentation [23], realistic image synthesis with stacked generative adversarial networks [24], and so on. In addition, GAN is also favored by researchers for image fusion. For example, Ma proposed Pan-GAN in remote sensing image fusion, which can obtain multi-spectral images of high-resolution by fusing panchromatic images and multispectral images of low-resolution [25], Li proposed coupled GAN with relativistic discriminators for infrared and visible image fusion, where the simple yet efficient relativistic discriminator is applied [26], and so on.

GAN, shown in Equation (1), is composed of two competing neural networks: a discriminator and a generator, where the G tries to generate data being close to the distribution $P_{data}$ of real data, and the discriminator D tries to distinguish between real data and data generated by the generator. During the two network confrontations, the generator uses the discriminator as the loss function and updates its parameters to produce more realistic-looking data. On the other hand, the discriminator updates its generator parameters to better identify false data from real data; it relies on the standard multilayer perceptron architecture to realize the network.

(1) $$\underset{G}{\min}\underset{D}{\max}V(D,G)=E_{x\sim P_{data}(x)}\left[\log D(x)\right]+E_{z\sim P_{z}(z)}\left[\log\left(1-D\left(G(z)\right)\right)\right]$$

Subsequently, convolutional operations are introduced into GAN and the deep convolutional GAN (DCGAN) is generated. Sometimes, the loss of GAN fluctuates because the generator and discriminator undermine each other’s learning. As a result, the progressive growing of GAN (ProGAN) is a network that stabilizes GAN training by increasing the resolution of the generated images. Because of its transformation architecture, self-attention GAN (SAGAN) has also become much popular in recent years. Instead of focusing on creating more realistic images and improving the ability of GAN to perform fine control over the generated images, StyleGAN can be employed with any GAN to produce better results.

## 3. The Proposed Method

In this section, we describe the proposed noisy SAR image fusion method based on NLM and GAN. NLM is introduced first. Then, the network architecture of the proposed method is given to make it clearer and more readable.

### 3.1. NLM

NLM consists of image blocking and similarity grouping. To balance the effect and efficiency of image blocking, we abandon high-level blocking methods such as methods for image edges or regional features and adopt the traditional fixed-size sliding window blocking method. Some classical methods mainly use a certain distance between image blocks as a similar measurement. For two image blocks, the smaller their distance value is, the more similar they are. Common distance calculation methods include the method based on singular value, Euclidean distance, $l_{p}-norm$ and so on. Euclidean distance is employed in this paper.

### 3.2. The Network of the Proposed Method

**Figure 1:**
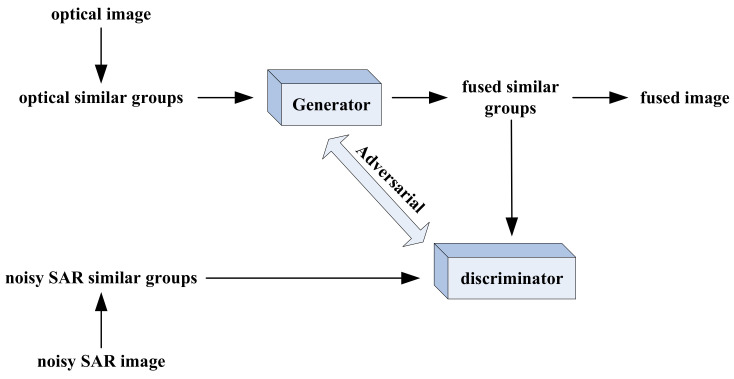
The network of the proposed method.

In order to retain the high spatial resolution of SAR image and color information of optical image more completely at the same time, we proposed a noisy SAR image fusion method based on NLM and GAN—shown in Figure 1. The similar image block groups obtained by NLM are fed into the generator at first, and the fused noise-free image $I_{f}$ can be generated by network training.

After that, the fused image and noisy SAR image $I_{s}$ are fed into the discriminator, which aims to determine whether the spatial resolution of fused and SAR images is consistent. More details about the network of the generator and discriminator are shown in Table 1.

At last, the loss function in Equation (2) of the proposed network contains two parts: the loss function of the generator $L_{G}$ in Equation (3), and the loss of the discriminator $L_{D}$ in Equation (5).

(2) $$Loss=aL_{G}+bL_{D}$$

where a and b are the weight factors that balance the contributions of the loss function of the generator and discriminator.

(3) $$L_{G}=L_{\mathrm{PSNR}}(I_{f})+l_{2}(I_{f},I_{o})=\frac{1}{n}\sum 1-\frac{\mathrm{PSNR}(B_{f})}{const}+{\left\|I_{f}-I_{o}\right\|}_{2}$$

where $\mathrm{PSNR}(B_{f})$ denotes peak signal-to-noise ratio (PSNR) of the fused image.

PSNR is often used to measure the noise level in an image:

(4) $$\mathrm{PSNR}(B_{f})=10\cdot \log_{10}\left[\frac{B_{\max}^{2}}{\mathrm{MSE}}\right]$$

The larger the PSNR, the better the image quality. const denotes a constant normalizing the value of PSNR and is set to 35, which means the maximum value of PSNR in an image, and the first term in Equation (3) can ensure that the fused image contains less noise; $l_{2}(I_{f},I_{o})$ denotes $l_{2}-norm$ loss of the optical image $I_{o}$ and fused image.

(5) $$L_{D}=L_{\mathrm{SSIM}}(I_{f},I_{s})+l_{2}(I_{f},I_{s})=\frac{1}{n}\sum 1-\mathrm{SSIM}(B_{f},B_{s})+{\left\|I_{f}-I_{s}\right\|}_{2}$$

where n denotes the total number of image blocks in source images. $\mathrm{SSIM}(B_{f},B_{s})$ denotes the structural similarity (SSIM) index of image blocks in the fused image and SAR image, which can be calculated as

(6) $$\mathrm{SSIM}(B_{f},B_{s})=\frac{2E[B_{f}]\cdot E[B_{s}]+C_{1}}{E[B_{f}^{2}]+E[B_{s}^{2}]+C_{1}}\cdot \frac{2\mathrm{cov}[B_{f},B_{s}]+C_{2}}{\mathrm{Var}[B_{f}]+\mathrm{Var}[B_{s}]+C_{2}}$$

where B represents the image block, $B_{f},B_{s}$ represent the fused image block and the SAR image block, respectively. $C_{1}$ and $C_{2}$ denote constants that are not zero. SSIM is a number greater than 0 and less than 1, which measures the correlation loss, brightness loss and contrast loss between source images and the fused image. The closer SSIM is to 1, the more similar the structure is.

## 4. Experimental Results and Analysis

### 4.1. Datasets and Parameter Settings

The training datasets were selected from SEN1–2 datasets [4], which contain more than twenty hundred thousand SAR-optical image pairs with the size of 256∗256 collected from across the globe and throughout all meteorological seasons. SAR images acquired by Sentinel-1 are polluted by speckle noise, whereas optical images acquired by Sentinel-2 are noise-free.

When NLM is employed to process the source images, the size of the image block is set to 32∗32, and the maximum image block in each similar group is 20. When training the proposed network, the generator and discriminator are optimized alternately, and we implemented our network in TensorFlow.

### 4.2. Compared Methods

To effectively evaluate the proposed noisy SAR image fusion method, in this section, we conducted the compared experiments by 8 representative image fusion methods, including image fusion based on guided filtering (GFF) [27], image fusion based on the sparse model (SR) [28], wavelet-based image fusion (DWT) [29], image fusion with deep convolutional neural network (CNN) [14], multi-scale weighted gradient-based fusion (MWGF) [30], image fusion based on multi-scale transform and sparse representation (MST-SR) [31], image fusion method in nonsubsampled Shearlet transform domain (NSST) [32], and a generative adversarial network for image fusion (GAN) [12]. Among all, MWGF and GFF belong to the methods based on spatial domain, while DWT and NSST are representative methods based on transform. For NSST, the employed fusion rule is “choose-max”. MST-SR is a hybrid method that combines diverse image fusion methods to implement better fused results, and NSCT is employed as multi-scale transform in this paper. CNN and GAN are popular methods based on deep learning. The codes of image fusion methods could be downloaded from links in their corresponding papers, and the parameters were set as recommended.

### 4.3. Valuable Metrics

To objectively evaluate different fusion methods, some objective metrics are employed to calculate the corresponding values of fused images, such as entropy (EN), average gradient (AVG), spatial frequency (SF), mutual information (MI), and Q^AB/F^ [33,34]. The larger these metric values are, the better the fused image is. The calculations of these metrics are as follows:

(1) EN

When we want to measure how much information an image contains, EN [33] is a good choice, and it can reflect the average amount of information contained in the fused image. It can be calculated by Equation (7).

(7) $$\mathrm{EN}=-\sum_{i=0}^{L-1}p_{i}\times \log_{2}p_{i}$$

where L denotes the total number of pixels in an image, and $p_{i}$ is the probability distribution for pixels in each gray level.

(2) AVG

The calculation of AVG is shown in Equation (8). We can evaluate the ability to vary tiny details and texture features in an image by the value of AVG.

(8) $$\mathrm{AVG}=\frac{1}{MN}\sum_{i=1}^{M}\sum_{j=1}^{N}\sqrt{\frac{\Delta I_{x}^{2}+\Delta I_{y}^{2}}{2}}$$

where $\Delta I_{x}=f(x,y)-f(x-1,y)$ and $\Delta I_{y}=f(x,y)-f(x,y-1)$.

(3) SF

SF is used to detect the total activity of a fused image in the spatial domain, which represents the ability to contrast small details. It can be calculated by Equation (9).

(9) $$\mathrm{SF}(i,j)=\sqrt{{\left(\mathrm{RF}\right)}^{2}+{\left(\mathrm{CF}\right)}^{2}}$$

where $\mathrm{RF}=\sqrt{\frac{1}{M\times N}\sum_{i=1}^{M}\sum_{j=2}^{N}{\left[f(x,y)-f(x,y-1)\right]}^{2}}$ represents row frequency while $\mathrm{CF}=\sqrt{\frac{1}{M\times N}\sum_{i=2}^{M}\sum_{j=1}^{N}{\left[f(x,y)-f(x-1,y)\right]}^{2}}$ represents column frequency.

(4) MI

MI represents the amount of information in a fused image from the source images, which also means the amount of information transferring from the source images to the fused image. More details about it are shown in Equation (10).

(10) $$\mathrm{MI}={\mathrm{MI}}_{\mathrm{AF}}+{\mathrm{MI}}_{\mathrm{BF}}$$

where ${\mathrm{MI}}_{\mathrm{AF}}=\sum_{f,a}P_{\mathrm{FA}}(f,a)\log\frac{P_{\mathrm{FA}}(f,a)}{P_{F}(f)P_{A}(a)}$ and ${\mathrm{MI}}_{\mathrm{BF}}=\sum_{f,b}P_{\mathrm{FB}}(f,b)\log\frac{P_{\mathrm{FB}}(f,b)}{P_{F}(f)P_{B}(b)}$. $P_{\mathrm{FA}}(f,a)$ and $P_{\mathrm{FB}}(f,b)$ denote the joint probability densities between the fused image F and the source images A,B, respectively, whereas $P_{A}(a)$ and $P_{B}(a)$ denote the probability densities of the source images.

### 4.4. Results and Analysis

#### 4.4.1. Experiments on SEN1–2

**Figure 2:**
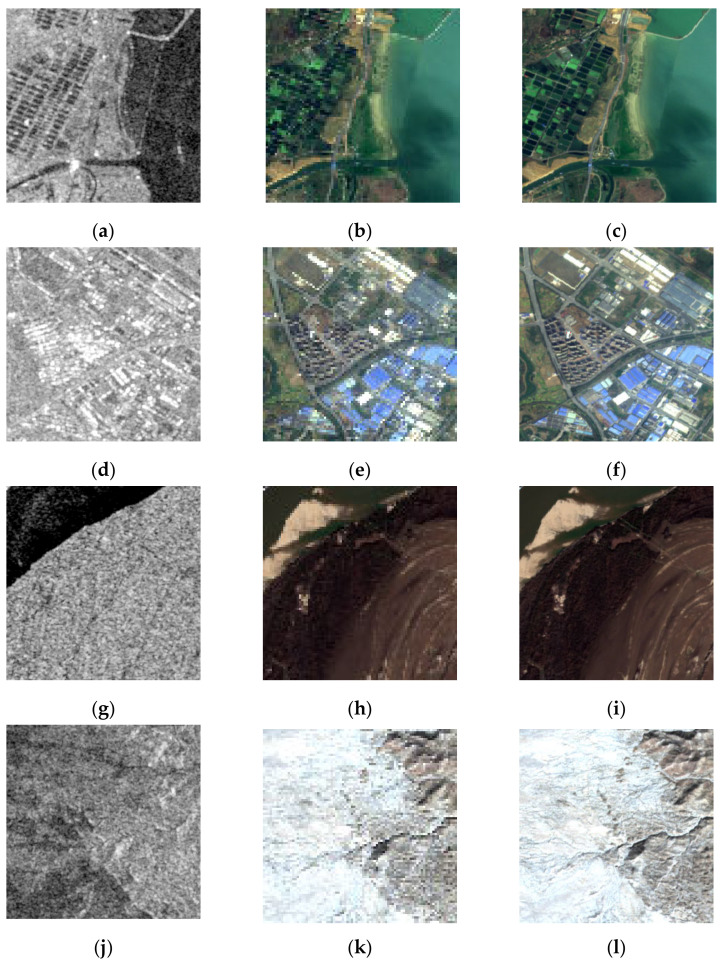
Results of our experiments using the SEN1–2 datasets by the proposed method. The first column: noisy synthetic aperture radar (SAR) images; the second column: optical images; the third column: fused images. (**a**–**c**) Group 1; (**d**–**f**) Group 2; (**g**–**i**) Group 3; (**j**–**l**) Group 4.

We randomly selected thirty thousand pairs of source images to perform the experiments using our proposed network. Twenty thousand of them were used for the training set, whereas the other ten thousand were used for the validation set. Figure 2 shows some examples from the SEN1–2 datasets, which include images sourced from different seasons. The first column in Figure 2 is the SAR source images with much speckle noise. The second column in Figure 2 is the optical source images with rich color information. The third column is the fused images by the proposed method. From the snow in Figure 2k, we can conclude that the image was taken in winter; however, it is hard to infer the season only from its corresponding SAR image, which has high resolution but no spectral information. By comparing the source images to the optical images, the spatial resolution of fused images has significantly improved, meaning the proposed method can extract spatial information from noisy SAR images and color information from optical images simultaneously.

**Figure 3:**
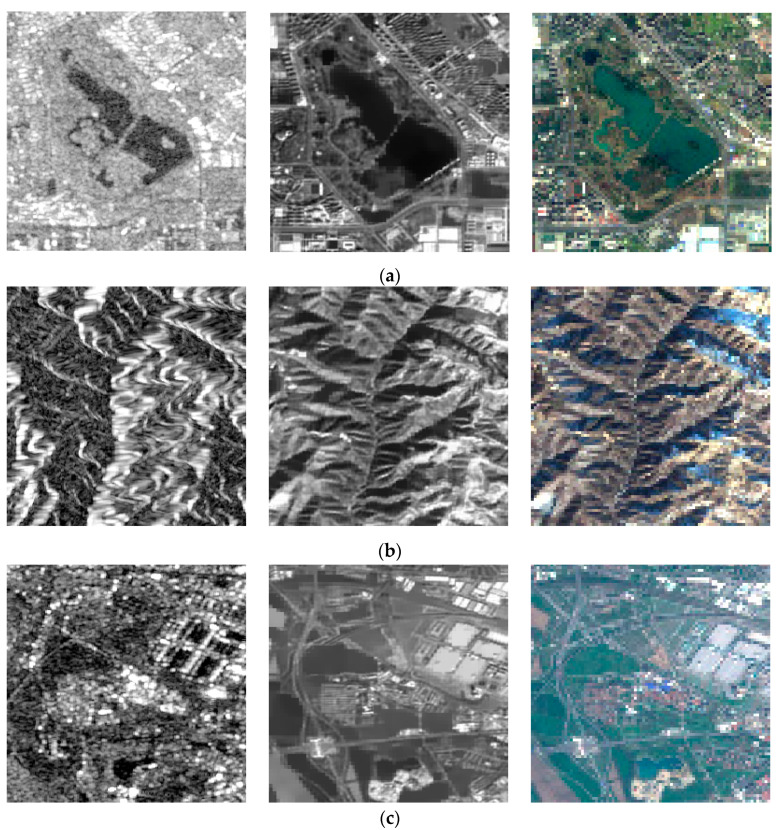

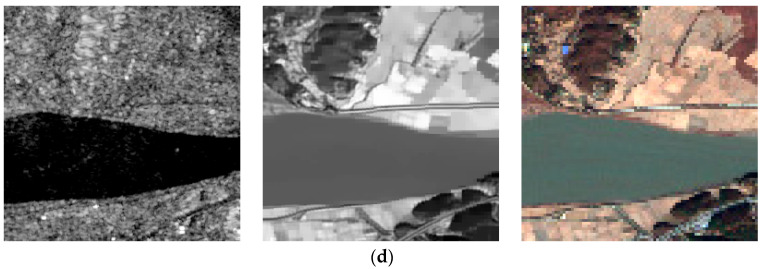
Compared source images in SEN1–2: the first column contains noisy SAR images, the second column contains de-noised SAR images, and the third column contains optical images. (**a**) Group 1; (**b**) Group 2; (**c**) Group 3; (**d**) Group 4.

**Figure 4:**
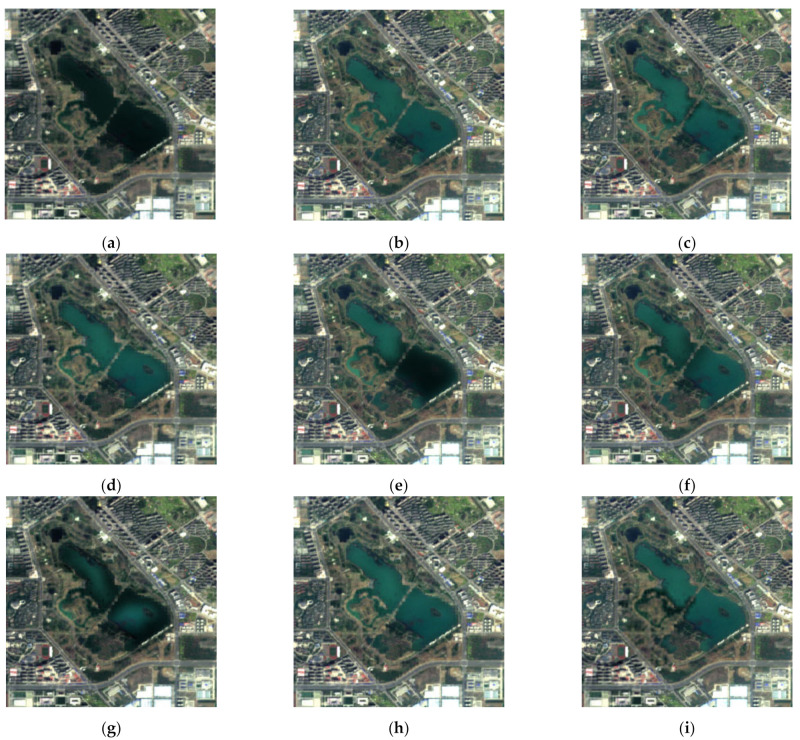
The fused images of Group 1 in Figure 3: (**a**) guided filtering (GFF); (**b**) sparse model (SR); (**c**) wavelet-based image fusion (DWT); (**d**) convolutional neural network (CNN); (**e**) multi-scale weighted gradient-based fusion (MWGF); (**f**) multi-scale transform and sparse representation (MST-SR); (**g**) nonsubsampled Shearlet transform domain (NSST); (**h**) generative adversarial network (GAN); (**i**) the proposed method.

**Figure 5:**
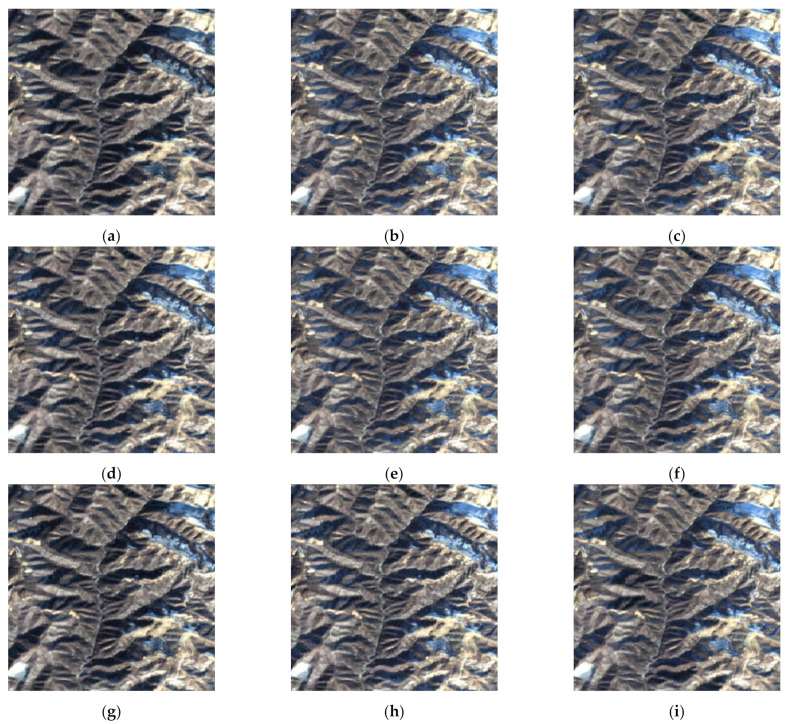
The fused images of Group 2 in Figure 3: (**a**) GFF; (**b**) SR; (**c**) DWT; (**d**) CNN; (**e**) MWGF; (**f**) MST-SR; (**g**) NSST; (**h**) GAN; (**i**) the proposed method.

**Figure 6:**
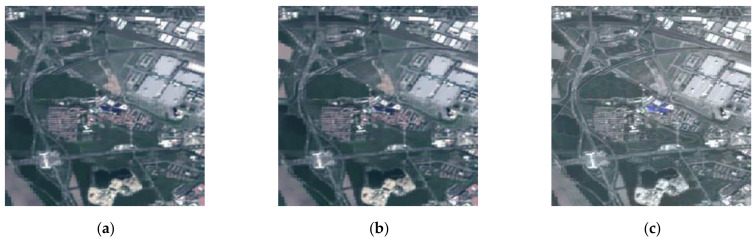

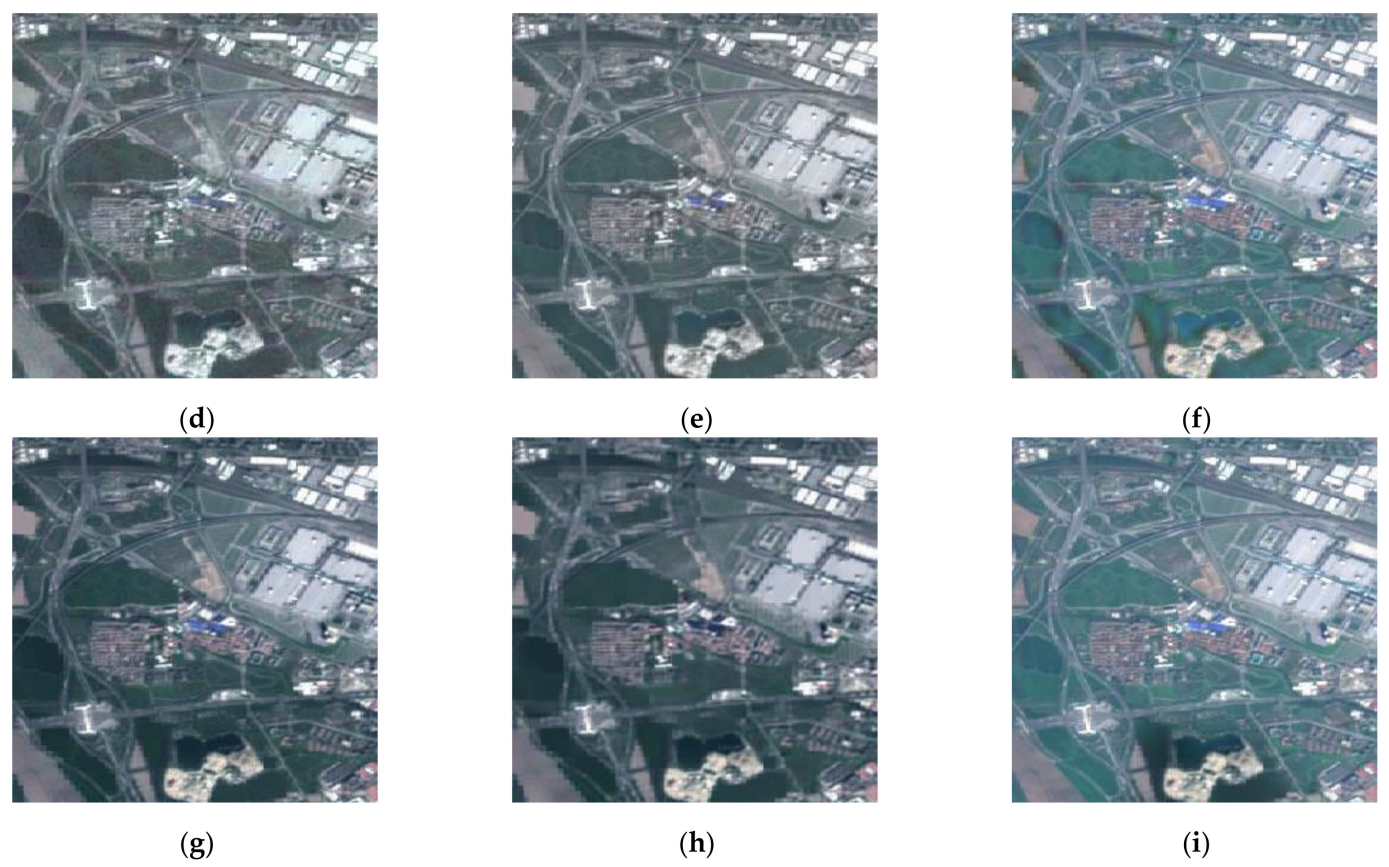
The fused images of Group 3 in Figure 3: (**a**) GFF; (**b**) SR; (**c**) DWT; (**d**) CNN; (**e**) MWGF; (**f**) MST-SR; (**g**) NSST; (**h**) GAN; (**i**) the proposed method.

**Figure 7:**
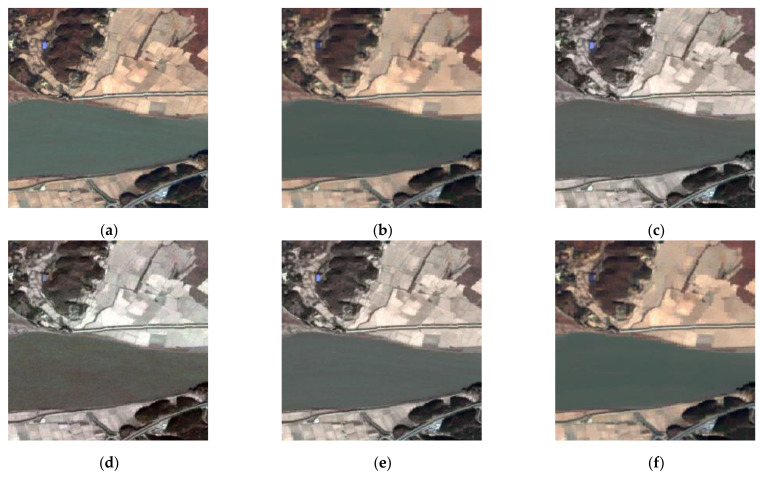

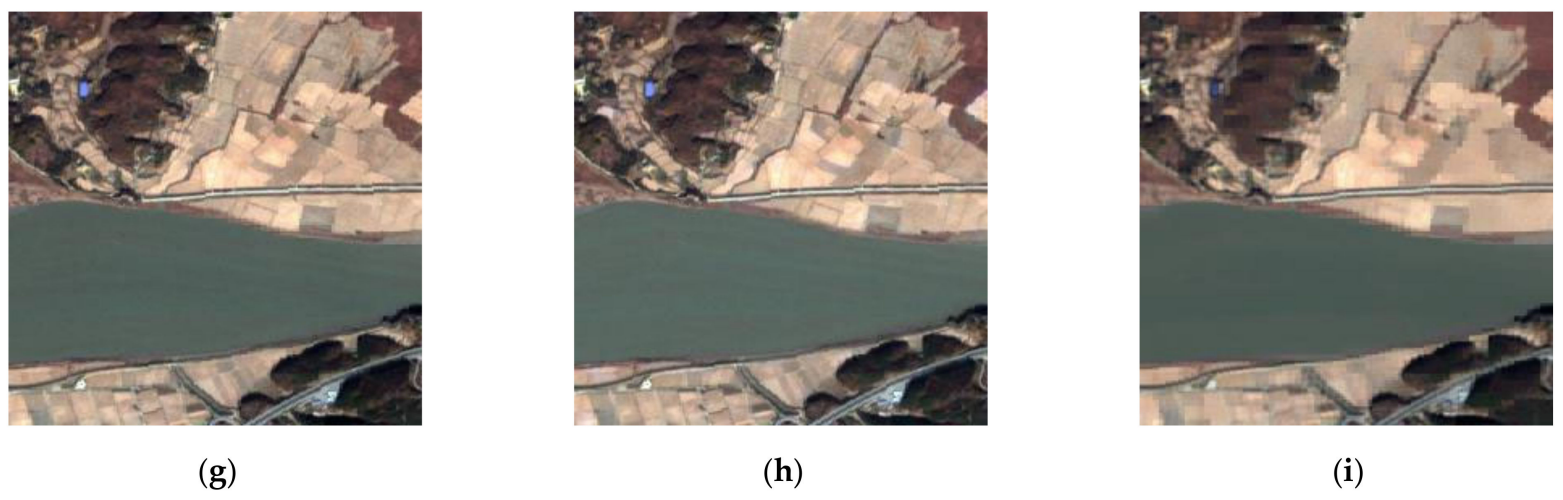
The fused images of Group 4 in Figure 3: (**a**) GFF; (**b**) SR; (**c**) DWT; (**d**) CNN; (**e**) MWGF; (**f**) MST-SR; (**g**) NSST; (**h**) GAN; (**i**) the proposed method.

In order to better verify the proposed method in this paper, we performed comparative experiments on SEN1–2, and the source images are shown in Figure 3. To ensure the fairness of the experiments, image de-noising by SAR-BM3D [20] was done on noisy images for subsequent image fusion in the compared methods. The fused images of Groups 1~4 in Figure 3 are shown in Figure 4, Figure 5, Figure 6 and Figure 7. We can see that there are significant color distortions and black areas of the green lake in Figure 4a,e,g. The fused images in Figure 6f,i are easier to distinguish due to the appropriate coloring. By carefully comparing the details of the fused images in Group 4, it is obvious that the fused image in Figure 7i by the proposed noisy SAR image fusion method has less speckle noise. All in all, from these fused images, it can be found that the proposed noisy SAR image fusion method not only has better fusion results but also is robust to image noise.

**Figure 8:**
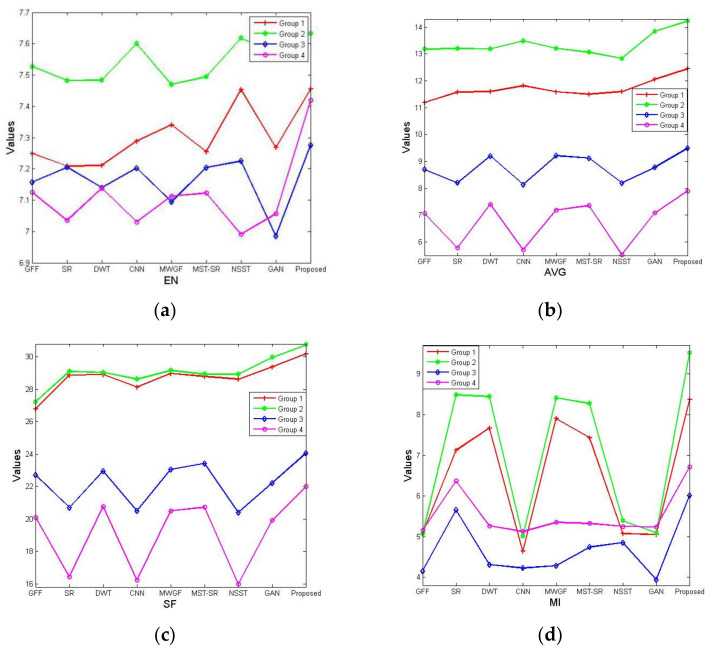
The valuable metrics of the fused images in Figure 3: (**a**) entropy (EN); (**b**) average gradient (AVG); (**c**) spatial frequency (SF); (**d**) mutual information (MI).

Moreover, valuable metrics are employed to evaluate the fused images. To make these values clearer, we show the valuable metrics in Figure 8. By comparing these values in Figure 8, we can conclude that the values in green are larger than the others, showing that the images in Group 2 are better than the other groups; however, when comparing the fused images in the same group by different fusion methods, it can be found that the values of the fused images by the proposed method are better, in general, indicating that the proposed method has more power on SAR image fusion. To further illustrate the generalization of the proposed method, we continue by testing 10 groups of images in SEN1–2 datasets randomly, and the average objective indicators are shown in Table 2. From Table 2, we can see that the proposed method can also obtain higher objective indicators.

#### 4.4.2. Experiments on Oslo City

**Figure 9:**
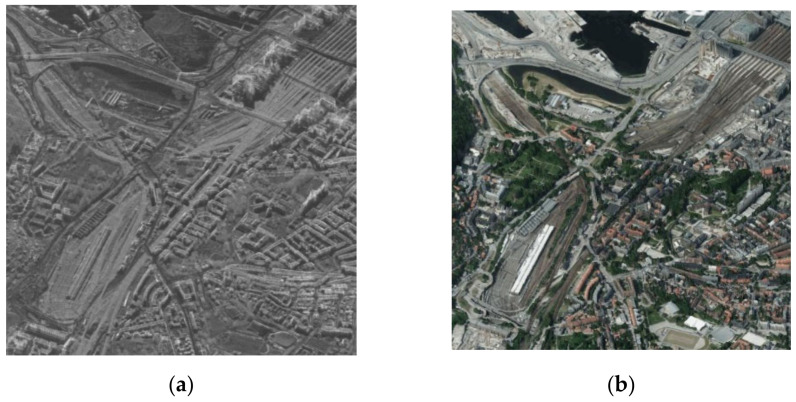
Oslo city: (**a**) SAR image; (**b**) optical image.

To verify the superiority and practicability of the proposed method, we compared experiments on source images of Oslo city, which are noise-free—see Figure 9.

**Figure 10:**
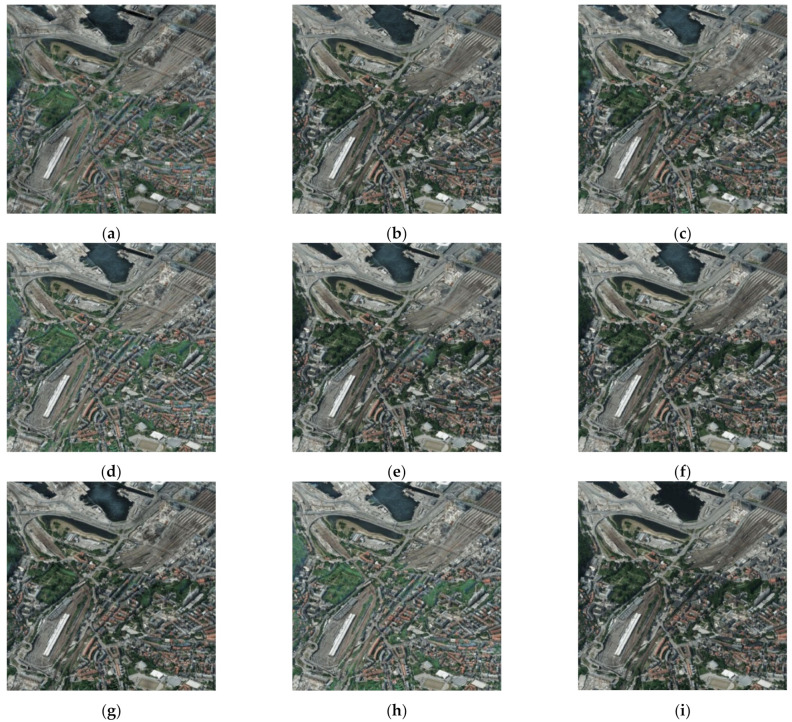
The fused images of Figure 8: (**a**) GFF; (**b**) SR; (**c**) DWT; (**d**) CNN; (**e**) MWGF; (**f**) MST-SR; (**g**) NSST; (**h**) GAN; (**i**) the proposed method.

The fused images of Figure 9 by different image fusion methods are shown in Figure 10. There is some color distortion in Figure 10a,d,h, where the color of the forests is bright green, whereas the color in the optical image is dark green. Besides, due to the multi-scale transformation of the images, some detailed information of source images was missed when fusing them using DWT, MST-SR, and NSST—see Figure 10c,f,g. By comparing the fused images in Figure 10, the fused image by the proposed method in Figure 10i has a better subjective effect and is more visually suitable to the human eye.

To evaluate the fused images in Figure 10 more objectively, objective metrics and computational time were employed, and the results are shown in Table 3. Although the values of the fused image by GAN are near to ours in terms of EN, AVG, and SF, the value of MI in Figure 10h is less than two. From Table 3, we can conclude that the fused image by the proposed method has better values of objective metrics and costs less time than most of the other methods.

## 5. Conclusions

Making full use of similar structural features in an image, a robust noisy SAR image fusion method based on NLM and GAN is proposed in this paper. Using the adversarial game of the generator and discriminator, a final fused noise-free image with high spatial resolution and color information can be obtained, where the optimization of the proposed network is realized by the constraint of the constructed loss function in this paper. By comparing our experiments with the state-of-the-art image fusion methods on the SEN1–2 datasets and Oslo city, we demonstrated that the proposed noisy SAR image fusion is robust to image noise and has a better fusion effect, which makes the images more suitable for the human eye. The fusion images obtained by the proposed method have less residual noise and color distortion. Meanwhile, it can retain the edge and texture details of the source images more effectively compared with other methods. In conclusion, the proposed method has excellent image de-noising performance and a better fusion effect. It is an excellent image fusion method for noisy SAR images and optic images, which can be extended to the multi-sensor image fusion.

However, the available datasets are limited and the trained model relies on the trained datasets, which means that our results are difficult to generalize. In the future, we will explore or create more datasets and test the noisy image fusion model further to improve our method and obtain more ideal effects.

## Figures and Tables

**Table 1:** The details of the generator and discriminator.

		Layer	Filter	Normalization	Activation
G	Encoder	En_1	5*5 Conv (n64)	BN	Leaky ReLU
		En_2	3*3 Conv (n128)	BN	Leaky ReLU
		En_3-En_5	3*3 Conv (n256)	BN	Leaky ReLU
	Decoder	De_1	3*3 Conv (n256)	BN	Leaky ReLU
		De_2	3*3 Conv (n128)	BN	Leaky ReLU
		De_3	5*5 Conv (n1)	-	Sigmoid
D		D_1	3*3 Conv (n64)	BN	Leaky ReLU
		D_2	3*3 Conv (n128)	BN	Leaky ReLU
		D_3	3*3 Conv (n256)	BN	Leaky ReLU
		D_4	3*3 Conv (n1)	-	Sigmoid

**Table 2:** Objective indicators of generalization on 10 test images from the SEN1–2 datasets.

	EN	AVG	SF	MI
GFF	7.2607 ± 0.0132	10.0256 ± 0.0636	24.6531 ± 0.2501	4.8751 ± 0.0082
SR	7.2251 ± 0.0161	9.6989 ± 0.1087	23.6590 ± 0.4291	6.8254 ± 0.0147
DWT	7.2426 ± 0.0114	10.1984 ± 0.0332	25.2251 ± 0.2619	6.3567 ± 0.0258
CNN	7.2675 ± 0.0503	9.9878 ± 0.1401	23.2157 ± 0.4069	4.6531 ± 0.0074
MWGF	7.2475 ± 0.0335	10.1538 ± 0.0408	25.3621 ± 0.2585	6.4256 ± 0.0361
MST-SR	7.2659 ± 0.0354	10.1596 ± 0.0395	25.0697 ± 0.2604	6.3751 ± 0.0292
NSST	7.3105 ± 0.1206	9.5635 ± 0.1537	23.2758 ± 0.4313	4.9253 ± 0.0102
GAN	7.2159 ± 0.0802	10.3756 ± 0.0819	25.5327 ± 0.3608	4.7754 ± 0.0146
Proposed	7.4225 ± 0.0205	10.8597 ± 0.0611	26.4568 ± 0.2503	7.5754 ± 0.0319

**Table 3:** The valuable metrics of the fused images in Figure 10.

	EN	AVG	SF	MI	Time(s)
GFF	7.1684	10.9946	25.8843	1.1225	0.864875
SR	7.3631	12.3016	30.3622	3.4416	77.549845
DWT	7.3449	12.1866	30.2316	3.8044	30.458764
CNN	7.3566	13.4386	32.2077	1.4334	141.987512
MWGF	7.4543	12.6963	31.2014	6.3148	3.648574
MST-SR	7.4561	12.7560	31.3776	6.6831	71.457981
NSST	7.4293	13.2218	31.7040	2.1016	4.987545
GAN	7.3815	13.8934	32.0352	1.5428	58.145457
Proposed	7.4694	14.7699	32.4543	7.6206	53.125794

## Data Availability

Not applicable.
